# A Sixth Modality of Infectious Disease: Contagious Cancer from Devils to Clams and Beyond

**DOI:** 10.1371/journal.ppat.1005904

**Published:** 2016-10-27

**Authors:** Michael J. Metzger, Stephen P. Goff

**Affiliations:** 1 Department of Biochemistry and Molecular Biophysics, Columbia University, New York, New York, United States of America; 2 Howard Hughes Medical Institute, New York, New York, United States of America; 3 Department of Microbiology and Immunology, Columbia University, New York, New York, United States of America; McGill University, CANADA

Infectious agents come in many forms, but they have been grouped into five distinct classes of agents: viruses, bacteria, fungi, parasites, and prions. Cancer is not normally on this list. Infectious agents like Human papilloma virus (HPV) or Human T-lymphotropic virus type 1 (HTLV-1) can cause cancers in infected hosts, but these cancers are generated within each new individual from oncogenic changes within the hosts’ own cells, and they stay within that individual. If cancer cells did travel from one individual to another, a normal immune system would be able to recognize them as foreign and reject them. Cancer is thus usually a self-limiting disease—it either regresses or it kills its host, and the death of the host marks the death of the cancer lineage.

But this is not always the case. Transmissible cancers have been identified as spreading within two vertebrates, dogs (*Canis lupus familiaris*) and Tasmanian devils (*Sarcophilus harrisii*), and more recently, multiple independent lineages of transmissible cancer have been found in four species of bivalves (Fig 1). This is an infectious modality that has significant effects on animals in both the terrestrial and marine environments, as well as in both vertebrates and invertebrates. While large-scale transmission of cancer has not been observed in humans, transmission between humans has been observed on a small scale in a number of circumstances, often in the context of immune suppression. With more research, more cases are likely to be found in humans as well as other animals.

**Fig 1:**
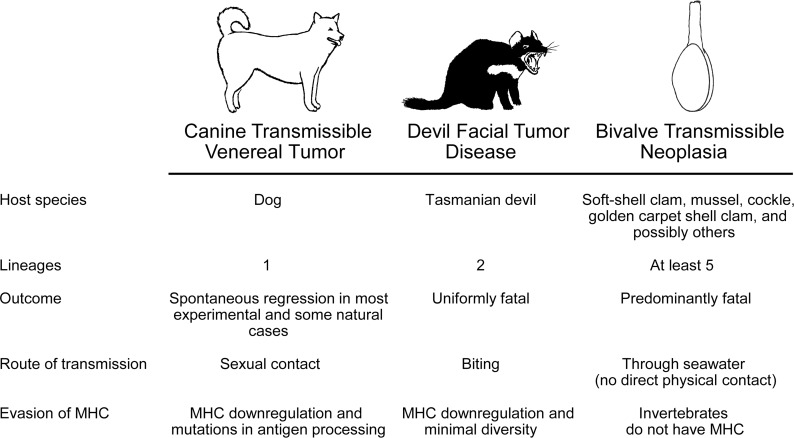
Comparison of the known lineages of infectious cancers in natural populations.

## Canine Transmissible Venereal Tumor

The first transmissible cancer to be identified was canine transmissible venereal tumor (CTVT), a solid tumor that spreads within populations of dogs through sexual contact. This was identified as a transmissible disease and was first experimentally transplanted from dog to dog in 1876 [1]. The etiology of the disease was initially uncertain, but many successful transplant experiments, common karyotypic rearrangements [2], and the finding of a unique integration of a LINE1 retrotransposon in front of the c-myc gene in all cases of CTVT [3] suggested that transmission occurs through transfer and replication of the cancer cells themselves, rather than through viral modification of cells in each new host. Analysis of dog leukocyte antigen alleles, polymorphic microsatellite loci, and mitochondrial DNA confirmed that this cancer is spread as a clonal transmissible cancer lineage [4,5]. Another unexpected feature of CTVT is that it has repeatedly acquired new mitochondrial genomes from its hosts throughout its evolution [6,7]. This suggests that asexual replication of cancer cells may lead to a Muller’s ratchet [8] process in mitochondria, where accumulation of mutations make cancers unsustainable in the long term without reintroduction of competent mitochondrial genomes.

CTVT spreads as a sexually transmitted infection in feral dog populations throughout the world, infecting animals in at least 90 countries on all continents except Antarctica [9]. An analysis of the CTVT genome concluded that the cells have been spreading as a transmissible cancer lineage for 10,000–12,000 years and likely arose from an early dog most closely related to Alaskan malamutes [10]. While experimentally transplanted CTVT usually regresses after a few months, naturally acquired disease does not always regress [2]. Thus, these cells have been evolving and spreading as an asexual parasitic organism that has outlived its original canine host by more than 500 generations.

## Tasmanian Devil Facial Tumor Disease

The first observation of a Tasmanian devil suffering from devil facial tumor disease (DFTD) was in 1996 [11]. DFTD is a solid facial tumor that spreads from animal to animal through physical contact when devils bite each other. It was identified as a transmissible cancer when a unique karyotypic rearrangement was found in tumors of multiple animals [12] and was confirmed after sequencing of the genomes of two isolates of DFTD [13]. The fatal disease continues to spread through the devil population and threatens them with extinction, although sequestered insurance populations and ongoing research on possible treatments may help maintain the species [14].

In a recent report, a second, apparently completely independent, lineage of DFTD (termed DFT2) was identified in a small number of animals, suggesting that if the conditions allow, transmissible cancers may arise multiple times [15]. Interestingly, the first lineage of DFTD identified (now termed DFT1) arose from a female devil, but DFT2 arose from a male. The first five cases of DFT2 were found in males, so it is possible that females have some ability to recognize the male DFT2 cells as nonself, but the numbers of cases currently reported are too low to confirm this.

## Bivalve Transmissible Neoplasias

Fatal leukemia-like neoplasias, called disseminated neoplasia or hemic neoplasia, have been reported in at least 15 different bivalve species [16,17]. They can occur at stable enzootic levels, but several epizootic events have been reported, including an outbreak of disease in soft-shell clams (*Mya arenaria*) on Prince Edward Island, Canada, where prevalences of >90% were recorded, and massive population loss was observed [17,18]. Analysis of the neoplastic cells in soft-shell clams revealed a dramatic amplification in the copy number of a retrotransposon [19], with identical integration sites in neoplastic cells from multiple animals. These data, along with analysis of microsatellites and mitochondrial DNA SNPs, showed that the etiologic agent of this disease is the neoplastic cell itself, as with CTVT and DFTD [20].

The finding of transmissible cancer in soft-shell clams and some evidence from disseminated neoplasia in mussels [21,22] suggested that the neoplasias of other bivalves could also be transmissible. Recently, disseminated neoplasia was analyzed from three additional bivalve species (the mussel, *Mytilus trossulus*; the cockle, *Cerastoderma edule*; and the golden carpet-shell clam, *Polititapes aureus*), and independent transmissible cancer cell lineages were observed in each species [23]. In both mussels and cockles, the cancer lineages arose from the corresponding host species, and in cockles, two apparently independent cockle-derived cancer lineages were found to be spreading through the population, as was found in Tasmanian devils. More unexpectedly, the cancer observed in *P*. *aureus* was found to be the result of a cross-species transmission, derived from a different, but related, species, *Venerupis corrugata*. Curiously, *V*. *corrugata* itself does not have a high incidence of the disease, despite living in the same area as *P*. *aureus*. As multiple lineages of transmissible cancers are spreading through multiple bivalve species, we call these diseases bivalve transmissible neoplasias (BTN). Since cancers from four bivalve species have been analyzed for transmissible cancer and all four were attributable to BTN lineages, it is reasonable to predict that disseminated neoplasia in other bivalves will be found to be due to BTN as well.

## Transmissible Cancers in Humans

Transmission of cancer from person to person is exceedingly rare. Any cancer cells that somehow find their way from a donor to a recipient would normally be recognized as foreign and rejected by a functional immune system. However, human-to-human transmission of cancer has been reported in several special settings [24], including transplant recipients acquiring cancer from tissue donors [25,26] and transfer of cancer from mother to fetus [27]. In a recent, remarkable case of immune suppression leading to transmission of cancer cells, an AIDS patient acquired neoplastic cells that derived from the cells of a dwarf tapeworm [28]. These are extreme cases in which both physical barriers and immune barriers are lowered.

Only a few cases of human cancer transmission without immune suppression or partially matched cells have been reported. There has been a case of a surgeon who accidentally introduced a cancer into his own hand during surgery [29], and at least one case of a needlestick accident leading to growth of a small nodule derived from a human adenocarcinoma cell line in an immunocompetent researcher [30]. In both cases, the tumors were excised and did not recur.

## Immune Responses to Contagious Cancer

In conventional cancers, the cancer cells must avoid recognition of neoantigens made by abnormal induction or mutation of host genes, but contagious cancers must also avoid recognition as cells from a foreign individual. The Major Histocompatibility Complex (MHC) genes in jawed vertebrates form a very strong (but not insurmountable) barrier to cancer allografts. Indeed, CTVT is known to down-regulate expression of MHC genes [31] and carries multiple mutations in genes involved in self-antigen presentation and apoptosis [32]. It has been suggested that lack of MHC diversity in devils contributes to the lack of host recognition of DFTD cells [33,34], but despite their low diversity, experimental allogeneic skin transplants in devils were recognized and rejected [35], and it has recently been shown that DFTD also down-regulates expression of MHC genes [36].

As in CTVT and DFTD, immune evasion plays a key role in the two major settings of human transmissible cancer mentioned above. Cancers transmitted during organ transplants can survive because the recipients are immunosuppressed, and cancers transmitted from mother to fetus are partially tolerated because the maternal cancer cells are half-matched to the fetus—and in at least one case, the neoplastic cells from the mother that grew in the infant had lost the MHC allele that was not shared with the infant [37]. Altogether, the data suggest that MHC-based self–nonself rejection is highly important in preventing transmissible cancers.

Bivalves are invertebrates and do not have MHC or any other known histocompatibility system, likely decreasing the barriers for evolution of transmissible cancer lineages. However, there is some evidence for species-specific restriction of BTN lineages, as most known lineages were derived from the original host species, and experimental transmission across species has not been successful [38,39]. Only one example of cross-species transmission from a related species has been observed, and in this case the original host species may have developed resistance to the lineage derived from it [23]. Currently, the mechanisms of these restrictions are unknown. It may resemble self–nonself recognition systems in vertebrates or may be an independent mechanism, as with the *BHF* gene involved in protecting tunicates from fusion with foreign colonies and stem cell parasitism [40].

## Cancer As an Infectious Organism

Clonal cancer lineages have spread far beyond their original hosts, replicating as asexual parasites that jump between individuals for decades (and even millennia, in the case of CTVT). It is, in fact, difficult to determine whether to describe these cells as “infecting” or “engrafting” their hosts, reflecting the fact that contagious cancers blur the lines between our normal definitions of cancer and infectious disease. They even stretch our understanding of species, as the living lineage of cells that form CTVT has not been a dog in 10,000 years. It is unlikely that cancer lineages can be considered to be new species, but regardless of terminology, their evolution and replication strategies are clearly distinct from those of their hosts. These contagious cancer lineages have developed a new infectious lifestyle separate from that of the organism from which they arose, and their new interactions provide strong selective pressures, which can affect evolution of both host and pathogen.

It is possible that the primary cancers that led to each of these transmissible cancer lineages could have been induced by a conventional pathogen, but so far there is no evidence of this. Some transmissible cancers may have been triggered by transposable elements; there is some indication of the involvement of transposons in CTVT [3] and soft-shell clam-transmissible neoplasia [19]. Regardless of their origin, all available evidence (including multiple genetic markers and full cancer genome sequencing of CTVT and DFTD) leads to the conclusion that the cancer cells themselves have become a pathogen, able to spread from individual to individual.

## Conclusions

Transmissible cancers appear to be limited by two main factors: immune recognition and physical barriers to the spread of cells. In a similar, but less extreme way, conventional cancers can also be thought of as infectious cells that have their own evolutionary pressures and strategies [41,42]. The evasion of immune recognition and the ability to metastasize have obvious parallels in the two main challenges for transmissible cancers.

We know that cases of cancer transmission do occur in humans in the special settings of organ transplant and maternal-to-fetal transfer. Transmission would not be likely to occur in immunocompetent people, but we would predict that it is conceivable that certain types of cancer could spread from person to person within immunosuppressed HIV-1-positive populations.

Currently, we know of transmissible cancers spreading through natural animal populations in six species across vertebrate and invertebrate organisms and in both terrestrial and marine ecosystems, and further study will likely uncover more examples. Despite recognition only recently as bona fide infectious diseases, transmissible cancers may have been developing, spreading, and producing a strong selective pressure on the evolution of organisms since the beginning of multicellularity.

## References

[1] Novinsky MA. Zur frage über die impfung der krebsigen geschwulste. Zentralbl Med Wiss. 1876;14:790–1.

[2] Cohen D. The canine transmissible venereal tumor: a unique result of tumor progression. Adv Cancer Res. 1985;43:75–112. 3887857 10.1016/s0065-230x(08)60943-4

[3] Katzir N, Rechavi G, Cohen JB, Unger T, Simoni F, Segal S, et al "Retroposon" insertion into the cellular oncogene c-myc in canine transmissible venereal tumor. Proc Natl Acad Sci U S A. 1985;82(4):1054–8. 2983328 10.1073/pnas.82.4.1054 PMC397192

[4] Murgia C, Pritchard JK, Kim SY, Fassati A, Weiss RA. Clonal origin and evolution of a transmissible cancer. Cell. 2006;126(3):477–87. 10.1016/j.cell.2006.05.051 16901782 PMC2593932

[5] Rebbeck CA, Thomas R, Breen M, Leroi AM, Burt A. Origins and evolution of a transmissible cancer. Evolution. 2009;63(9):2340–9. 10.1111/j.1558-5646.2009.00724.x 19453727

[6] Rebbeck CA, Leroi AM, Burt A. Mitochondrial capture by a transmissible cancer. Science. 2011;331(6015):303 10.1126/science.1197696 21252340

[7] Strakova A, Ni Leathlobhair M, Wang GD, Yin TT, Airikkala-Otter I, Allen JL, et al Mitochondrial genetic diversity, selection and recombination in a canine transmissible cancer. Elife. 2016;5. 10.7554/eLife.14552 PMC4869914 27185408

[8] Muller HJ. The Relation of Recombination to Mutational Advance. Mutat Res. 1964;106:2–9. 14195748 10.1016/0027-5107(64)90047-8

[9] Strakova A, Murchison EP. The changing global distribution and prevalence of canine transmissible venereal tumour. BMC Vet Res. 2014;10:168 10.1186/s12917-014-0168-9 25186078 PMC4152766

[10] Murchison EP, Wedge DC, Alexandrov LB, Fu B, Martincorena I, Ning Z, et al Transmissible dog cancer genome reveals the origin and history of an ancient cell lineage. Science. 2014;343(6169):437–40. 10.1126/science.1247167 24458646 PMC3918581

[11] Hawkins CE, Baars C, Hesterman H, Hocking GJ, Jones ME, Lazenby B, et al Emerging disease and population decline of an island endemic, the Tasmanian devil Sarcophilus harrisii. Biological Conservation. 2006;131(2):307–24.

[12] Pearse AM, Swift K. Allograft theory: transmission of devil facial-tumour disease. Nature. 2006;439(7076):549 10.1038/439549a 16452970

[13] Murchison EP, Schulz-Trieglaff OB, Ning Z, Alexandrov LB, Bauer MJ, Fu B, et al Genome sequencing and analysis of the Tasmanian devil and its transmissible cancer. Cell. 2012;148(4):780–91. 10.1016/j.cell.2011.11.065 22341448 PMC3281993

[14] McCallum H, Jones M, Hawkins C, Hamede R, Lachish S, Sinn DL, et al Transmission dynamics of Tasmanian devil facial tumor disease may lead to disease-induced extinction. Ecology. 2009;90(12):3379–92. 20120807 10.1890/08-1763.1

[15] Pye RJ, Pemberton D, Tovar C, Tubio JM, Dun KA, Fox S, et al A second transmissible cancer in Tasmanian devils. Proc Natl Acad Sci U S A. 2016;113(2):374–9. 10.1073/pnas.1519691113 26711993 PMC4720317

[16] Carballal MJ, Barber BJ, Iglesias D, Villalba A. Neoplastic diseases of marine bivalves. J Invertebr Pathol. 2015;131:83–106. 10.1016/j.jip.2015.06.004 26146225

[17] Barber BJ. Neoplastic diseases of commercially important marine bivalves. Aquatic Living Resources. 2004;17(4):449–66.

[18] Muttray A, Reinisch C, Miller J, Ernst W, Gillis P, Losier M, et al Haemocytic leukemia in Prince Edward Island (PEI) soft shell clam (Mya arenaria): spatial distribution in agriculturally impacted estuaries. Sci Total Environ. 2012;424:130–42. 10.1016/j.scitotenv.2012.02.029 22425172

[19] Arriagada G, Metzger MJ, Muttray AF, Sherry J, Reinisch C, Street C, et al Activation of transcription and retrotransposition of a novel retroelement, Steamer, in neoplastic hemocytes of the mollusk Mya arenaria. Proc Natl Acad Sci U S A. 2014;111(39):14175–80. 10.1073/pnas.1409945111 25201971 PMC4191779

[20] Metzger MJ, Reinisch C, Sherry J, Goff SP. Horizontal transmission of clonal cancer cells causes leukemia in soft-shell clams. Cell. 2015;161(2):255–63. 10.1016/j.cell.2015.02.042 25860608 PMC4393529

[21] Moore JD, Elston RA, Drum AS, Wilkinson MT. Alternate pathogenesis of systemic neoplasia in the bivalve mollusc Mytilus. J Invertebr Pathol. 1991;58(2):231–43. 1783779 10.1016/0022-2011(91)90067-z

[22] Vassilenko EI, Muttray AF, Schulte PM, Baldwin SA. Variations in p53-like cDNA sequence are correlated with mussel haemic neoplasia: A potential molecular-level tool for biomonitoring. Mutat Res. 2010;701(2):145–52. 10.1016/j.mrgentox.2010.06.001 20541620

[23] Metzger MJ, Villalba A, Carballal MJ, Iglesias D, Sherry J, Reinisch C, et al Widespread transmission of independent cancer lineages within multiple bivalve species. Nature. 2016;534(7609):705–9. 10.1038/nature18599 27338791 PMC4939143

[24] Lazebnik Y, Parris GE. Comment on: 'guidelines for the use of cell lines in biomedical research': human-to-human cancer transmission as a laboratory safety concern. Br J Cancer. 2015;112(12):1976–7. 10.1038/bjc.2014.656 25584491 PMC4580382

[25] Sala-Torra O, Hanna C, Loken MR, Flowers ME, Maris M, Ladne PA, et al Evidence of donor-derived hematologic malignancies after hematopoietic stem cell transplantation. Biol Blood Marrow Transplant. 2006;12(5):511–7. 10.1016/j.bbmt.2006.01.006 16635786

[26] Myron Kauffman H, McBride MA, Cherikh WS, Spain PC, Marks WH, Roza AM. Transplant tumor registry: donor related malignancies. Transplantation. 2002;74(3):358–62. 12177614 10.1097/00007890-200208150-00011

[27] Tolar J, Neglia JP. Transplacental and other routes of cancer transmission between individuals. J Pediatr Hematol Oncol. 2003;25(6):430–4. 12794519 10.1097/00043426-200306000-00002

[28] Muehlenbachs A, Bhatnagar J, Agudelo CA, Hidron A, Eberhard ML, Mathison BA, et al Malignant Transformation of Hymenolepis nana in a Human Host. N Engl J Med. 2015;373(19):1845–52. 10.1056/NEJMoa1505892 26535513

[29] Gartner HV, Seidl C, Luckenbach C, Schumm G, Seifried E, Ritter H, et al Genetic analysis of a sarcoma accidentally transplanted from a patient to a surgeon. N Engl J Med. 1996;335(20):1494–6. 10.1056/NEJM199611143352004 8890100

[30] Gugel EA, Sanders ME. Needle-stick transmission of human colonic adenocarcinoma. N Engl J Med. 1986;315(23):1487 10.1056/NEJM198612043152314 3785302

[31] Siddle HV, Kaufman J. Immunology of naturally transmissible tumours. Immunology. 2015;144(1):11–20. 10.1111/imm.12377 25187312 PMC4264906

[32] Decker B, Davis BW, Rimbault M, Long AH, Karlins E, Jagannathan V, et al Comparison against 186 canid whole-genome sequences reveals survival strategies of an ancient clonally transmissible canine tumor. Genome Res. 2015;25(11):1646–55. 10.1101/gr.190314.115 26232412 PMC4617961

[33] Cheng Y, Sanderson C, Jones M, Belov K. Low MHC class II diversity in the Tasmanian devil (Sarcophilus harrisii). Immunogenetics. 2012;64(7):525–33. 10.1007/s00251-012-0614-4 22460528

[34] Siddle HV, Sanderson C, Belov K. Characterization of major histocompatibility complex class I and class II genes from the Tasmanian devil (Sarcophilus harrisii). Immunogenetics. 2007;59(9):753–60. 10.1007/s00251-007-0238-2 17673996

[35] Kreiss A, Cheng Y, Kimble F, Wells B, Donovan S, Belov K, et al Allorecognition in the Tasmanian devil (Sarcophilus harrisii), an endangered marsupial species with limited genetic diversity. PLoS One. 2011;6(7):e22402 10.1371/journal.pone.0022402 21811598 PMC3141043

[36] Siddle HV, Kreiss A, Tovar C, Yuen CK, Cheng Y, Belov K, et al Reversible epigenetic down-regulation of MHC molecules by devil facial tumour disease illustrates immune escape by a contagious cancer. Proc Natl Acad Sci U S A. 2013;110(13):5103–8. 10.1073/pnas.1219920110 23479617 PMC3612627

[37] Isoda T, Ford AM, Tomizawa D, van Delft FW, De Castro DG, Mitsuiki N, et al Immunologically silent cancer clone transmission from mother to offspring. Proc Natl Acad Sci U S A. 2009;106(42):17882–5. 10.1073/pnas.0904658106 19822752 PMC2764945

[38] Mateo DR, MacCallum GS, Davidson J. Field and laboratory transmission studies of haemic neoplasia in the soft-shell clam, Mya arenaria, from Atlantic Canada. J Fish Dis. 2015. 10.1111/jfd.12426 26687447

[39] Kent ML, Wilkinson MT, Drum AS, Elston RA. Failure of transmission of hemic neoplasia of bay mussels, Mytilus trossulus, to other bivalve species. J Invertebr Pathol. 1991;57(3):435–6. 2066579 10.1016/0022-2011(91)90148-j

[40] Voskoboynik A, Newman AM, Corey DM, Sahoo D, Pushkarev D, Neff NF, et al Identification of a colonial chordate histocompatibility gene. Science. 2013;341(6144):384–7. 10.1126/science.1238036 23888037 PMC3810301

[41] Greaves M, Maley CC. Clonal evolution in cancer. Nature. 2012;481(7381):306–13. 10.1038/nature10762 22258609 PMC3367003

[42] Nowell PC. The clonal evolution of tumor cell populations. Science. 1976;194(4260):23–8. 959840 10.1126/science.959840

